# Multifaceted property tailoring of polyamide 6 by blending miscible and immiscible components: ternary blends of polyamide 6/polyethylene terephthalate/phenol novolac†

**DOI:** 10.1039/d0ra02344b

**Published:** 2020-04-17

**Authors:** Takayuki Hirai, Yusaku Onochi, Jumpei Kawada

**Affiliations:** Material and Processing Department, Polymer Processing and Mechanics Laboratories, Toyota Central R&D Laboratories, Inc. 41-1 Yokomichi Nagakute 480-1192 Japan hirai@mosk.tytlabs.co.jp; Lightweight Material Development Group, Organic Material Dept., Organic Material Engineering Div., Toyota Motor Corporation 1 Toyota-cho Toyota Aichi 471-8572 Japan

## Abstract

Ternary polymer blends comprising miscible and immiscible components are examined to improve the mechanical properties of polyamide 6 (PA6) under humid and high-temperature conditions. Miscible polymers increase the glass transition temperature (*T*_g_), owing to their strong inter-molecular interactions, while phase-separated immiscible polymers reinforce the physical properties of PA6 as filler materials. Ternary blends exhibit these combined miscible and immiscible component contributions. Thus, in this study, ternary blends comprising PA6, polyethylene terephthalate (PET, immiscible component), and phenol novolac (PN, miscible component) are prepared by melt mixing. The PA6 stiffness in the water-absorbed state is reinforced by PET. Moreover, the proposed PA6/PET/PN ternary blends exhibit higher *T*_g_ values and lower water absorption rates than those of the PA6/PET binary blend, owing to the PN contribution. The PET and PN contributions are achieved independently and can be controlled *via* the composition ratios of the component polymers. Multifaceted property tailoring is thus demonstrated.

## Abstract 2

Properties of polyamide 6 (PA6) under hygrothermal conditions are improved by blending polyethylene terephthalate (PET, immiscible) and phenol novolac (PN, miscible).

## Graphical abstract

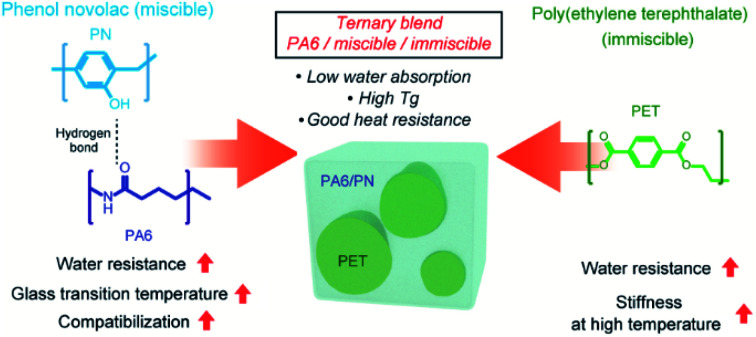

## Introduction

Polyamide 6 (PA6) has been widely applied to industrial products such as electronics, packaging, and automobile parts. The superior characteristics of PA6 are based on the contribution of the inter-molecular hydrogen bonding between the amide groups. However, these hydrogen bonds also cause hygroscopicity, a crucial defect in polyamides.^1,2^ Water molecules form hydrogen bonds with amide groups and act as plasticizers.^1^ As a result, under humid conditions, the glass transition temperature (*T*_g_) of PA6 decreases from ∼50 °C in the dry state to ∼−10 °C in the water-absorbed state (Fig. S1†). Thus, it is difficult to apply PA6 to products that are used in humid and high-temperature conditions.

Polymer blending is an attractive method for industry because it enables the fabrication of tailored polymeric materials with a cost-effective process.^3–5^ Polymer blends can be divided into two types, namely miscible and immiscible systems.^6^ Moreover, the loading of miscible and immiscible components modifies the material properties in different and complementary ways. In miscible systems, the component polymers generate attractive inter-molecular interactions, and their blends display homogeneous morphologies and single *T*_g_ values. Thus, by blending with a miscible component, *T*_g_ shifting can be achieved without the influence of the phase morphology. On the other hand, in immiscible systems, the component polymers are phase-separated and behave independently; thus, immiscible polymer blends display multiple *T*_g_ values of the component polymers. Immiscible components can therefore modify the physical properties as filler materials, while the properties of the immiscible blends are influenced by their phase morphology.

Various polymer blends have been proposed to improve the mechanical properties of PA6 under humid and high-temperature conditions.^7–21^ Owing to the small mixing enthalpy of macromolecules, almost all polymer combinations are immiscible^5^ and thus, previous studies have mainly conducted PA6 polymer blending with immiscible components.^7–14^ Blending high-*T*_g_ polymers, such as poly(phenylene sulfide),^7,8^ poly(phenylene oxide),^9–11^ and polycarbonate (PC)^12–14^ with PA6 are representative approaches to increase the PA6 stiffness at high temperatures. In these blends, immiscible components in the glassy state act as reinforcing fillers of PA6 in the rubbery state at temperatures ranging between the *T*_g_ of PA6 and that of the immiscible component. On the other hand, phenol-containing polymers, such as phenol novolac (PN) and PA6 blends, have been reported as miscible systems.^15–21^ PN forms strong hydrogen bonds with PA6, which contribute to the miscibility of such blends.^16,18^ Miscible components can increase the *T*_g_ of PA6, and we previously reported that high-molecular-weight PN efficiently increases the *T*_g_ of PA6.^20^ Furthermore, it has been reported that PN prevents water absorption of PA6 by the contribution of strong hydrogen bonding.^15,18–20^

Although miscible and immiscible components are known to enhance different PA6 properties, to the best of our knowledge, no studies on PA6 ternary blends comprising both immiscible and miscible components have been conducted to date.

**Fig. 1:**
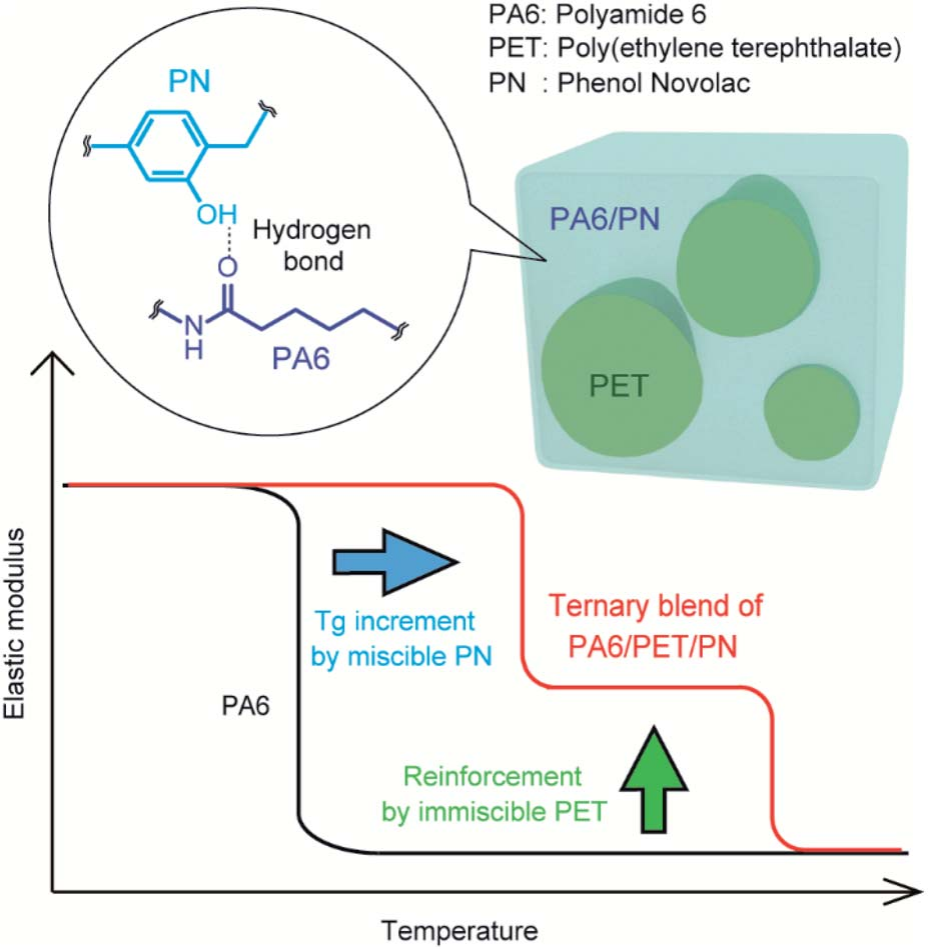
Conceptual image of the enhancement of the elastic modulus of PA6 by blending with PET (immiscible) and PN (miscible). The elastic modulus at high temperatures above the glass transition temperature (*T*_g_) of PA6 is reinforced by the immiscible component, while the *T*_g_ of PA6 itself can be increased by the miscible component.

Such ternary blends have the potential to achieve the combined benefits of components with different miscibilities; moreover, multifaceted properties can be tailored by controlling their composition ratios. Herein, we propose a PA6/polyethylene terephthalate (PET)/PN ternary blend predicted to display the combined properties of high stiffness at high temperatures and high *T*_g_ values *via* the contributions of their immiscible PET and miscible PN components, respectively (conceptual image, Fig. 1).

In this study, the PA6, PET, and PN blends were prepared by melt mixing, and the properties of the ternary blends were confirmed by evaluating the water absorption rates, crystallization behavior, viscoelasticity, static mechanical properties, and phase morphologies.

## Experimental

### Sample preparation

Standard-grade PA6 (A1030BRL, Unitika, Japan), injection-molding-grade PET (TRN-8550FF, Teijin Limited, Japan), and heat-resistant-grade PN (PAPS PN70, Asahi Yukizai, Japan, *M*_n_ = 2200) were employed in this study. All the polymers were vacuum-dried prior to melt mixing. Melt mixing was conducted using an internal mixer (Labo Plastomill, Toyo Seiki Seisaku-sho, Japan) with a cylinder temperature of 250 °C (PA6/PN binary blend) and 270 °C (other blends), rotation speed of 100 rpm, and mixing time of 5 min.

The sample designations and composition ratios of the prepared blends are listed in Table 1.

**Table 1:** Sample designations and constituent weight fractions of polyamide 6 (PA6), polyethylene terephthalate (PET), and phenol novolac (PN)

Sample designation	PA6 (wt%)	PET (wt%)	PN (wt%)
PA6/PN20	80	0	20
PA6/PET	50	50	0
PA6/PET/PN10	45	45	10
PA6/PET/PN20	40	40	20

### Water absorption test

Homo PA6, homo PET, and the melt-mixed samples were melt-pressed into rectangular specimens (50 mm × 10 mm × 2 mm) using a laboratory press at the same temperatures used for melt-mixing. The specimens were then cooled by a second laboratory press to 150 °C to promote their crystallization by slow cooling. First, the specimens were vacuum-dried at 80 °C for 12 h and weighed. Next, the specimens were immersed in distilled water at 23 °C, until they all reached the equivalent state, and then weighed. Finally, the water absorption rate (*χ*_w_) was calculated using eqn (1):1
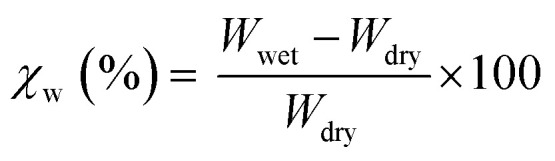
where *W*_dry_ and *W*_wet_ are the weights of the specimens just after vacuum drying and after reaching the equivalent state in water, respectively.

### Differential scanning calorimetry (DSC)

The crystallization and melting behavior of homo PA6, homo PET, and the melt-mixed samples were evaluated using DSC (Q1000, TA Instruments, USA). Samples of ∼10 mg were cut from the melt-mixed samples, placed in aluminum pans, and then sealed. The measurements were carried out in the range of 20–250 °C (PA6, PA6/PN20) and 280 °C (others) at a heating rate of 10 °C min^−1^ and cooling rate of 20 °C min^−1^ under nitrogen flow. Subsequently, the degrees of crystallinity (*χ*_c_) of the component polymers were calculated from eqn (2), while the melting enthalpies (Δ*H*_m_) and temperatures (*T*_m_) were obtained by analyzing the DSC first heating scan curves. The employed melting enthalpies of 100% crystallized (Δ*H*_0_) PA6 and PET were 190 (ref. 22 and 23) and 121 J g^−1^,^24^ respectively.2
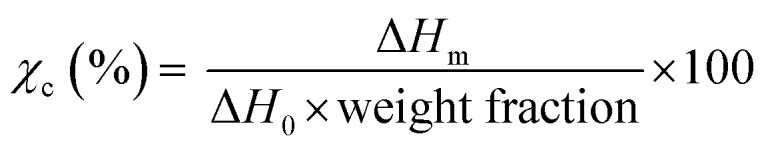


### Dynamic mechanical analysis (DMA) at water-absorbed state

Homo PA6, homo PET, and the melt-mixed samples were melt-pressed into rectangular specimens (35 mm × 5 mm × 0.5 mm) using the same procedure as that described for the water absorption tests. Prior to measurement, the specimens were immersed in distilled water at 23 °C until they all reached the equivalent state. DMA was then carried out using a dynamic mechanical analyzer (DVA-225, ITK, Japan) in the temperature range −100 to 150 °C at a heating rate of 5 °C min^−1^ and frequency of 10 Hz.

### Flexural test at water-absorbed state

The specimens that underwent the water absorption test were then subjected to flexural testing at the water-absorbed state. The flexural tests were carried out using a universal testing machine (model 5566, Instron, USA) in a thermostatic room at 23 ± 2 °C. The supporting distance and test speed were set to 32 mm and 1 mm min^−1^, respectively and measurements were conducted with *n* = 5.

### Scanning electron microscopy (SEM)

The cryo-fractured surfaces of the polymer blends were fabricated by fracturing the rectangular specimens (50 mm × 10 mm × 2 mm) in liquid nitrogen. The fractured surfaces were sputter-coated with platinum and observed with a scanning electron microscope (S-3600N, Hitachi, Japan) at an accelerating voltage of 15 kV.

### Transmission electron microscopy (TEM)

The rectangular specimens (50 mm × 10 mm × 2 mm) were cryo-microtomed into thin films. The PA6 in the samples was stained with ruthenium tetroxide and the samples were then observed with a transmission electron microscope (H-7650, Hitachi, Japan) at an accelerating voltage of 100 kV.

## Results

### Water absorption test

**Fig. 2:**
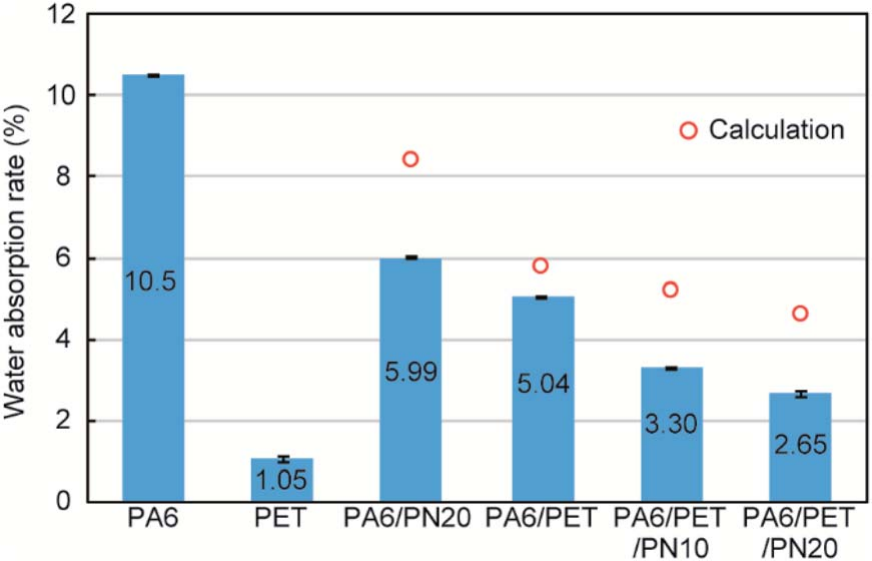
Water absorption rates of the different specimens. The error bar represents the standard deviation (*n* = 3). The red circles represent the water absorption rates calculated based on the water absorption rates and weight fractions of polyamide 6 (PA6) and polyethylene terephthalate (PET).

The water absorption test results are summarized in Fig. 2. The expected water absorption rates of the polymer blends, illustrated as red circles in Fig. 2, were calculated using eqn (3):

3 *χ*_calc_ = *χ*_PA6_*φ*_PA6_ + *χ*_PET_*φ*_PET_

where *χ*_calc_ represents the calculated water absorption rates; *χ*_PA6_ and *χ*_PET_ are the measured water absorption rates of homo PA6 and homo PET, respectively; and *φ*_PA6_ and *φ*_PET_ are the weight fractions of PA6 and PET, respectively.

In these calculations, we ignored the water absorption rate of PN because it could not be determined accurately, owing to its extremely high hygroscopic behavior. Thus, the true values of the calculated water absorption rates of PA6/PN20 and the ternary blends were larger than the values presented in Fig. 2.

Homo PA6 presented a high water absorption rate of >10%, while homo PET showed almost 1/10 of the value of PA6. PA6/PN20 exhibited a lower water absorption rate than PA6, which was lower than the calculated value due to the strong intermolecular interaction between PA6 and PN. PA6/PET showed almost half of the value of PA6 due to the lower hygroscopicity of PET. The observed differences between the measured and calculated values of the water absorption rate of PA6/PET were attributed to the labyrinth effect of the phase-separated morphology of this blend. However, the effect of PET, which is the immiscible component, on the reduction in water absorption could be estimated from the weight fraction. The PA6/PET/PN ternary blend displayed a combined behavior of PA6/PN and PA6/PET. The ternary blend showed a lower water absorption than that estimated from the weight fraction, owing to the intermolecular interactions between PA6 and PN. These results indicate that PN prohibited the water absorption of PA6 in the PA6/PET/PN blend as well as the PA6/PN binary blends.

### DSC

**Fig. 3:**
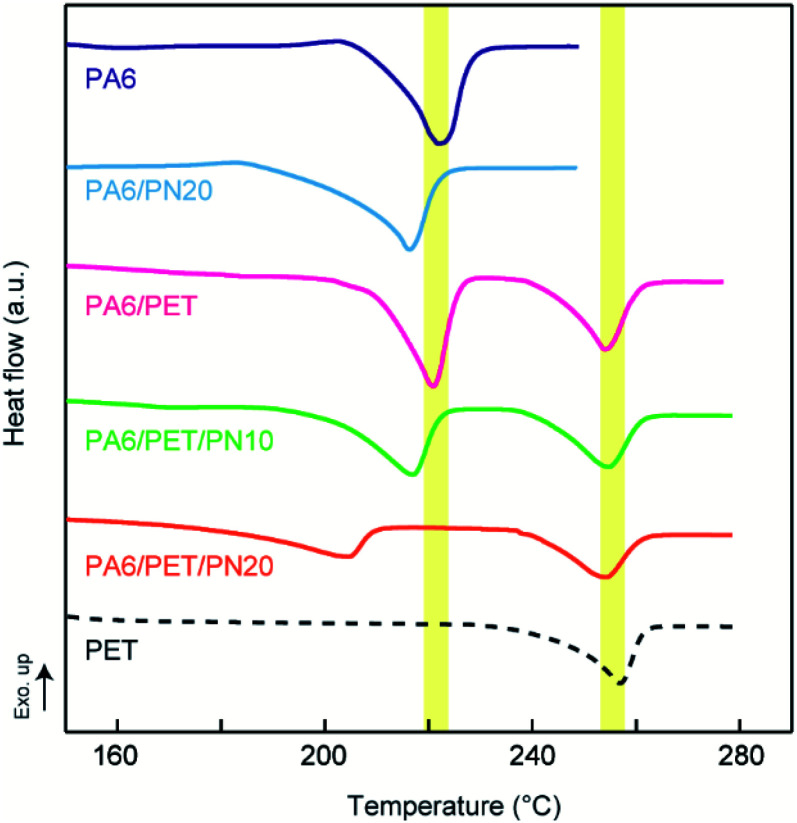
Differential scanning calorimetry (DSC) curves of the first heating scan. The melting enthalpies of polyamide 6 (PA6) and polyethylene terephthalate (PET) were calculated by integration of the endothermic peak, while the melting temperatures of PA6 and PET were determined as the peak temperatures of the endothermic peaks.

The DSC first heating scans are displayed in Fig. 3, while the melting temperatures and crystallinities of PA6 and PET in the blends are listed in Table 2.

**Table 2:** Melting temperatures and crystallinities of polyamide 6 (PA6) and polyethylene terephthalate (PET) in the tested blends calculated based on the differential scanning calorimetry (DSC) first heating scans

Sample designation	Melting temperature (°C)		Crystallinity (%)	
	PA6	PET	PA6	PET
PA6	221	—	37.2	—
PET	—	257	—	35.0
PA6/PN20	216	—	36.7	—
PA6/PET	221	253	33.4	34.4
PA6/PET/PN10	217	254	32.0	36.5
PA6/PET/PN20	204	253	22.8	39.9

For PA6, the melting temperatures were lowered by PN loading, which indicates miscibility of PA6 and PN.^25^ Particularly, compared to that of the other blends, the PA6 in PA6/PET/PN20 exhibited a markedly lower crystallinity. It has been reported that the miscible component disrupts the crystallization of PA6 (ref. 26 and 27) and excess PN loading results in low PA6 crystallinity. In PA6/PN20 and PA6/PET/PN10, which comprise almost the same composition ratios of PA6 and PN, the melting temperatures of PA6 are almost the same, suggesting that the PN in the ternary blend sufficiently modified the PA6 component. On the other hand, the crystallization behavior of PET is barely influenced, and the differences are caused by the phase morphology changes and heterogeneous nucleation initiated at the interface between PET and PA6.^28,29^

### DMA at the water-absorbed state

**Fig. 4:**
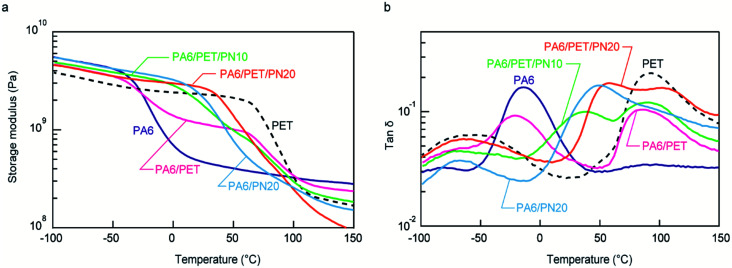
Viscoelastic behavior of the polymer blends and homo polymers. Temperature dependencies of (a) the storage moduli and (b) tan *δ*. The measurements were conducted in the water-absorbed state.

Fig. 4a indicates the temperature dependencies of the storage moduli in the water-absorbed state. PA6 was softened at ∼−20 °C because it was plasticized by the water molecules; thus, in the water-absorbed state, it exhibited a low storage modulus of the rubbery state at temperatures >0 °C. In a binary blend of PA6/PN20, the softening point is positively shifted from homo PA6 due to its miscibility. As a result, PA6/PN20 maintains a high storage modulus of the glass state at approximately 20 °C, while the storage modulus in the high temperature region (above 50 °C) is not enhanced. The modulus of the PA6/PET blend was an average of the homo PA6 and homo PET moduli at all measured temperature regions, owing to their immiscibility. Consequently, the storage modulus of PA6/PET was higher than that of PA6 in the range −20–100 °C because within this temperature range, the PET in the glassy state reinforces the PA6 in the rubbery state. As shown above, PN and PET caused different effects on the viscoelastic behavior of PA6.

In the ternary blends, PN increased the softening point corresponding to PA6; thus, the ternary blends maintained higher moduli in the glassy state at higher temperatures than those observed with homo PA6 and PA6/PET. PA6/PET/PN20 presented a softening point above room temperature; however, compared to those of the other blends, this blend displayed lower storage moduli at temperatures >100 °C. This was attributed to a decrease in the crystallinity of PA6, as illustrated in Table 2. The temperature dependencies of tan *δ* are displayed in Fig. 4b. The PA6/PET blend presented a bimodal peak at almost the same temperature as that of homo PA6 and homo PET, which is a typical behavior of immiscible polymer blends.^6^ In the ternary blends, the peak temperature of PA6 was shifted from −10 °C to 40 °C and 55 °C in PA6/PET/PN10 and PA6/PET/PN20, respectively, due to PN loading.

### Flexural test at water-absorbed state

**Fig. 5:**
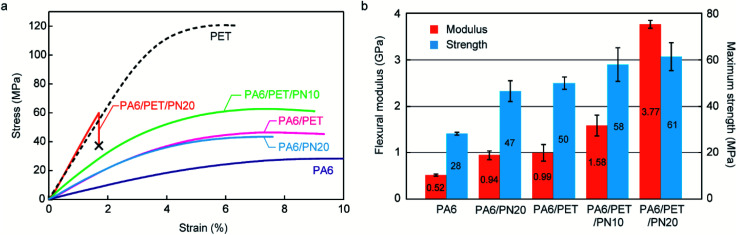
Results of the flexural test at the water-absorbed state. (a) Stress–strain curves of the polymer blends and homo polymers in the water-absorbed state. The point marked with an *X* represents the point of fracture of the specimen. (b) Flexural moduli and maximum strengths of homo PA6 and the polymer blends. The flexural moduli were defined as the slope of the stress–strain curves in the strain range 0.5–2.5%. Error bar represents the standard deviation (*n* = 5).

The stress–strain curves obtained by flexural testing are illustrated in Fig. 5a, while the flexural moduli and maximum strength of each specimen, obtained from the stress–strain curves, are illustrated in Fig. 5b. At the water-absorbed state, PA6 displayed poor mechanical properties in rubbery state. Both PA6/PN20 and PA6/PET exhibited an improved flexural modulus and maximum strength over those of homo PA6; however, the improved mechanical properties of PA6/PN20 and PA6/PET were achieved by different mechanisms. In PA6/PN20, the *T*_g_ of the PA6 component was increased by blending PN, and the high stiffness of PA6/PN20 was achieved because PA6/PN20 exhibits the glassy state at the measurement condition (23 ± 2 °C). On the other hand, the novelty of PA6/PET is caused by the reinforcement of PET in the glassy state. The ternary blends achieved these combined benefits, and PA6/PET/PN10 included an extent of glassy PA6 at room temperature (Fig. 4); thus, it displayed superior mechanical properties to those of PA6/PET. PA6/PET/PN20 also displayed high moduli in the glassy state, owing to the high *T*_g_ of PA6 in PA6/PET/PN20, which is above room temperature. However, embrittlement, a disadvantage of excess PN loading to PA6,^20^ was observed.

### SEM

**Fig. 6:**
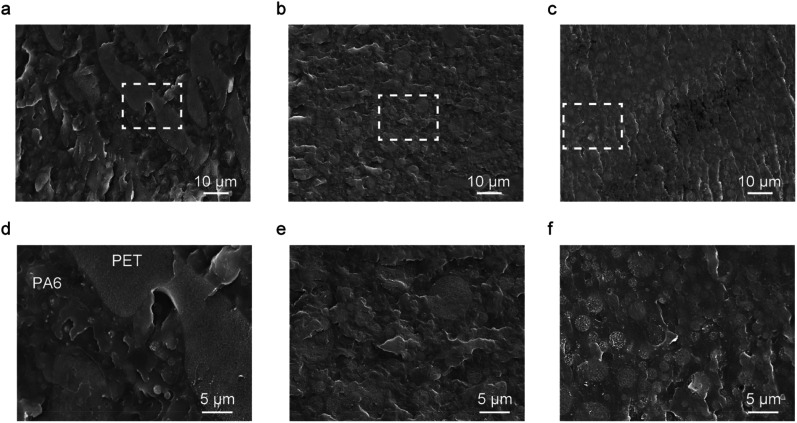
Scanning electron micrographs of (a and d) polyamide 6/polyethylene terephthalate (PA6/PET), (b and e) PA6/PET/phenol novolac (PN)10, and (c and f) PA6/PET/PN20. (a–c) Low-magnified images of the polymer blends and (d–f) high-magnified images of the areas selected in the white dashed boxes.

The phase morphologies of the polymer blends are illustrated in Fig. 6. The PA6/PET blend displayed a large-scale phase separation of >100 μm, with the island phase considered to be PET. This is because equal weight fractions of PA6 and PET correspond to 55 vol% PA6 and 45 vol% PET, owing to the different densities of PA6 (1.13 g cm^−3^)^30^ and PET (1.38 g cm^−3^).^31^ The size of the island phases was decreased by PN loading, and the ternary blends presented a smoother surface than that of the PA6/PET binary blend. We recently reported on the catalytic effect of PN on the exchange reaction between PA6 and PC^32^ and thus, in this study, we predicted that PN would promote an exchange reaction between PA6 and PET. We considered that miniaturization of the PET phase is caused by the PA6/PET copolymers as compatibilizers, which are generated by the catalyzed exchange reaction.

### TEM

**Fig. 7:**
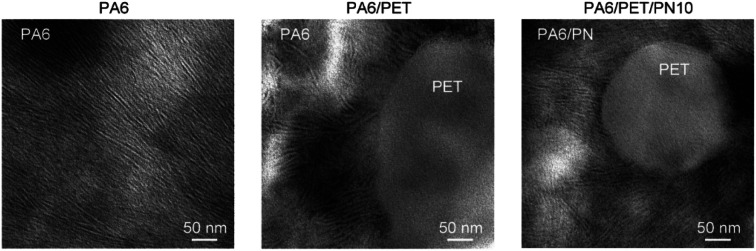
Transmission electron micrographs of polyamide (PA)6 and the polymer blends. Lamellar stacks of PA6 can be observed because of the staining treatment.

The TEM images are presented in Fig. 7. The lamellar stack of PA6 crystals is observed in homo PA6 and the PA6 phase in the polymer blends. The PA6 phases in PA6/PET and PA6/PET/PN10 were nearly identical, and the phase separation of the PN phase in PA6 was not observed in this view field. This observation supports the schematic image of the phase morphology of the ternary blends in Fig. 1.

### Generality and expansion of the approach

The results revealed that the contributions of the miscible and immiscible components can be achieved independently. Multifaceted property tailoring and superior properties of the ternary blends under humid and high-temperature conditions were also demonstrated in PN composition ratios ranging between 5 and 20 wt% (Fig. S2†). However, in dry state, the contributions of the ternary blends were limited because PA6 displays a high *T*_g_ of ∼50 °C (Fig. S3†). Moreover, loading an excess amount of PN (>20 wt%) causes some drawbacks such as low crystallinity and ductility.

Herein, we only reported the results from the PA6/PET/PN ternary blends; however, we also confirmed that other polyesters, such as poly(butylene terephthalate) (PBT) and poly(ethylene naphthalate) (PEN), can be alternatives to PET. The PA6/PBT/PN and PA6/PEN/PN blends presented reinforced moduli at high temperatures and increased *T*_g_ as was observed for PA6/PET/PN (Fig. S4 and S5†). Compatibilization by PN loading was also observed in the PA6/PBT/PN and PA6/PEN/PN ternary blends (Fig. S6†). Furthermore, as an extension of our new blending concept, quaternary polymer blending of PA6, PET, PN, and poly(ether imide) (PEI) was conducted. In these quaternary blends, the *T*_g_ values of the PET component could be increased by PEI blending, owing to their miscibility (Fig. S7 and S8†).^33^

## Conclusions

In this study, ternary blends of PA6, immiscible PET, and miscible PN were proposed to improve the properties of PA6 under humid and high-temperature conditions. Immiscible PET reinforces PA6 at temperatures in the range −20–100 °C. This is because in this temperature range, PET in the glassy state acts as a filler material for PA6 in the rubbery state. PN contributes toward the increased *T*_g_ and decreased water absorption of PA6. The effects of PET and PN appeared independently, indicating that the properties of the ternary blends can be widely tailored by controlling their composition ratios. This study successfully demonstrates the effectiveness of blending both miscible and immiscible components, and the search for other attractive polymer combinations ensues.

## Conflicts of interest

There are no conflicts to declare.

## Supplementary Material

RA-010-D0RA02344B-s001
