# Supplementary material for: Multifaceted property tailoring of polyamide 6 by blending miscible and immiscible components: ternary blends of polyamide 6/polyethylene terephthalate/phenol novolac
Source: RSC Adv. 2020 Apr 17;10(26):15132–8. doi: 10.1039/d0ra02344b (PMC9052217; doi:10.1039/d0ra02344b)
Supplement: RA-010-D0RA02344B-s001 [file RA-010-D0RA02344B-s001.pdf]

Electronic Supplementary Material (ESI) for RSC Advances.
This journal is © The Royal Society of Chemistry 2020

Supporting Information

Multifaceted Property Tailoring of Polyamide 6 by Blending Miscible and Immiscible components: Ternary Blends of Polyamide 6/Polyethylene Terephthalate/Phenol Novolac

Takayuki Hirai[a,*], Yusaku Onochi[b], Jumpei Kawada[a]

[a]Material and Processing Department, Polymer Processing and Mechanics Laboratories, Toyota Central R&D Laboratories, Inc., 41-1 Yokomichi, Nagakute 480-1192, Japan

[b]Lightweight Material Development Group, Organic Material Dept., Organic Material Engineering Div., Toyota Motor Corporation, Toyota-cho, Toyota, Aichi, Japan

*Corresponding Author. **Email:** hirai@mosk.tytlabs.co.jp

Abbreviations

PA6: Polyamide 6, PBT: poly(butylene terephthalate), PEN: poly(ethylene naphthalate), PEI: poly(ether imide), PN: phenol novolac

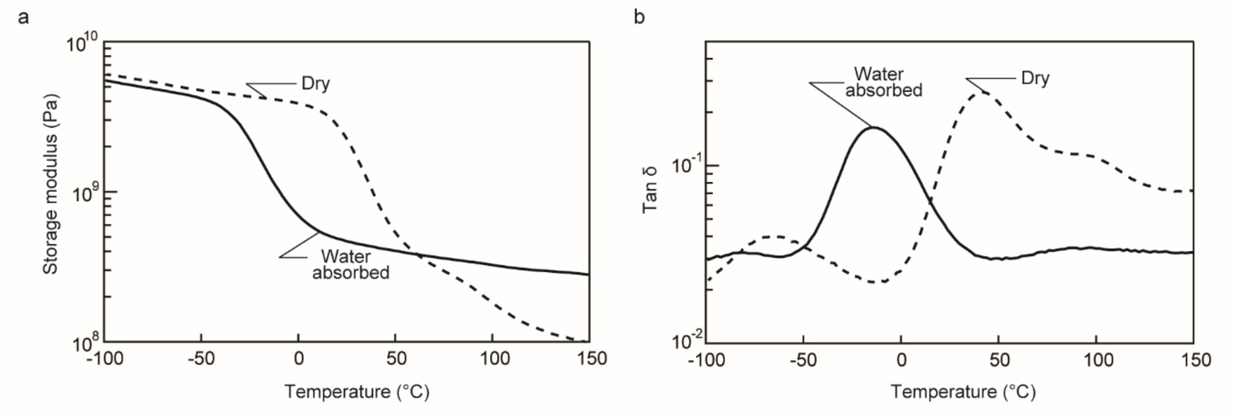

Figure S1. Temperature dependencies of (a) the storage modulus and (b) tan δ of PA6 in dry state after vacuum drying and water-absorbed state after reaching equivalent state in water. The water molecules act as a plasticizer and the glass transition temperature (T_g) of PA6 is low-temperature shifted by water absorption.

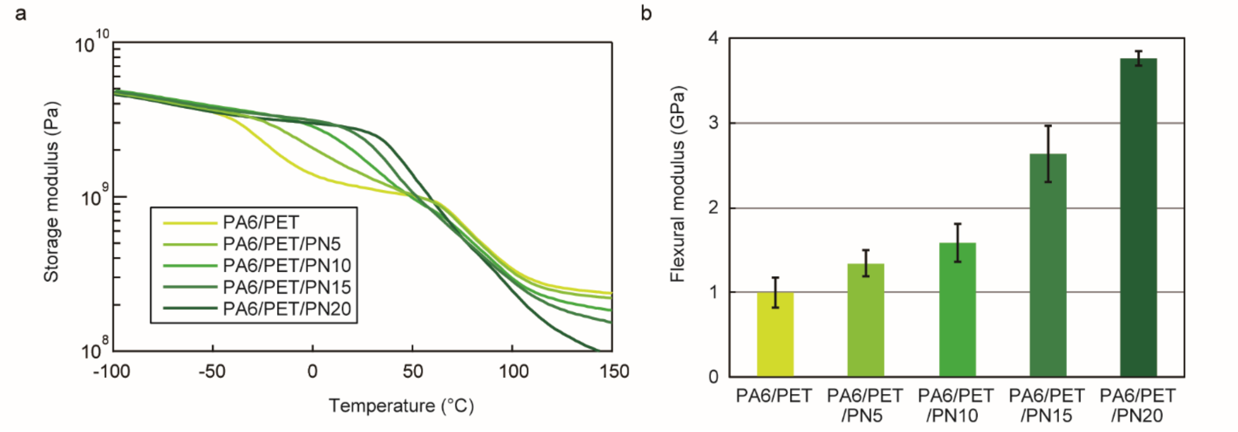

Figure S2. (a) Temperature dependencies of the storage modulus and (b) flexural moduli of PA/PET and ternary blends in water-absorbed state (PA6/PET/PN5:PA6/PET including 5 wt% PN and PA6/PET/PN15: including 15 wt% PN.)

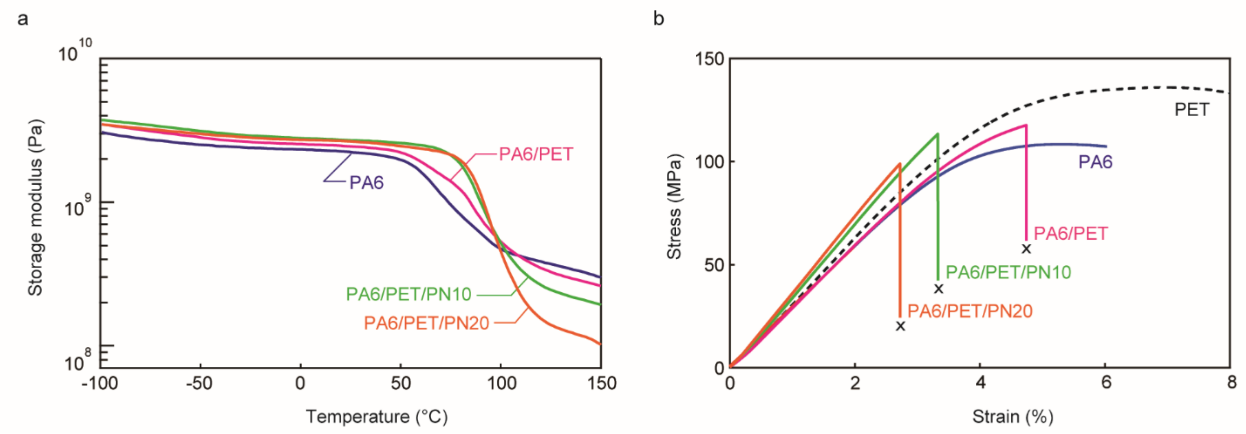

Figure S3. (a) Viscoelastic behavior and (b) stress–strain curves of PA6 and the polymer blends, which were obtained from vacuum-dried specimens.

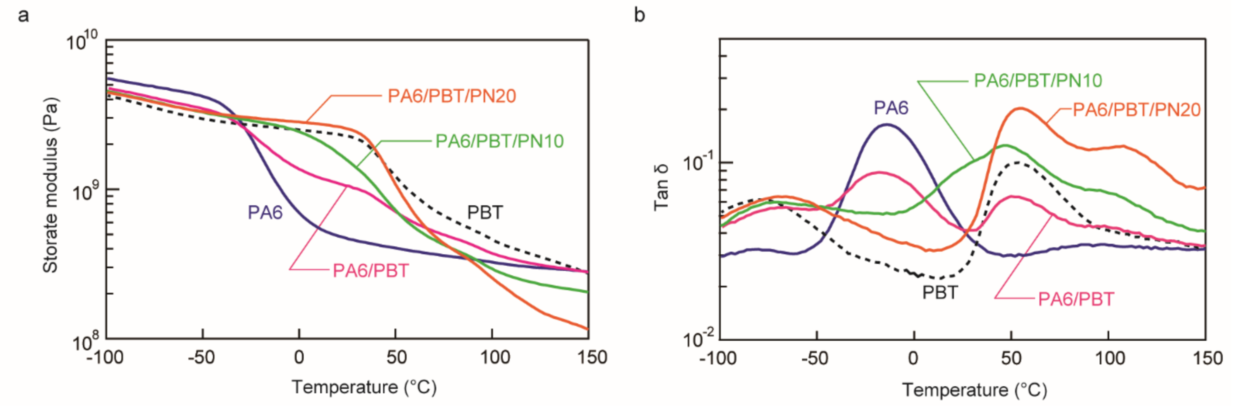

Figure S4. Viscoelastic behavior of homo PA6, homo PBT, and the polymer blends in water-absorbed state. Temperature dependencies of (a) the storage modulus and (b) tan δ.

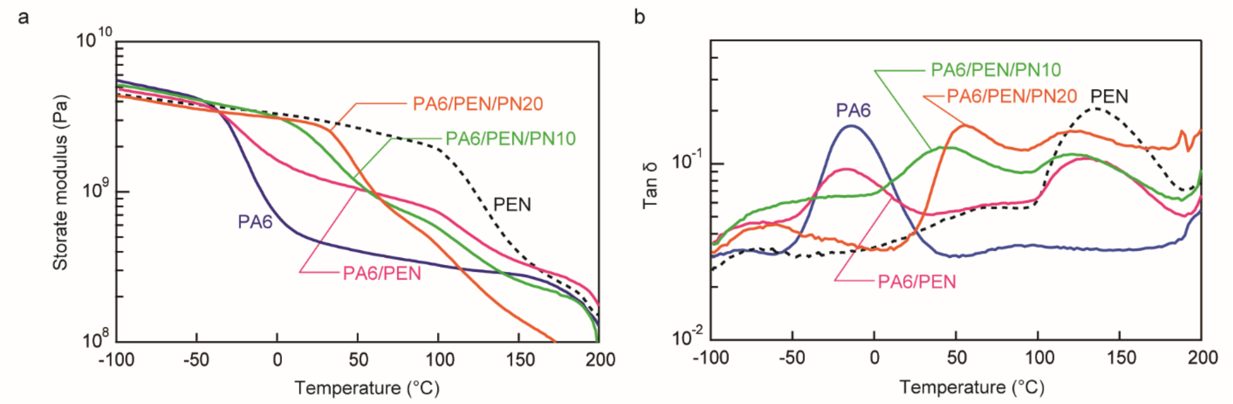

Figure S5. Viscoelastic behavior of homo PA6, homo PEN, and the polymer blends in water-absorbed state. Temperature dependencies of (a) the storage modulus and (b) tan δ.

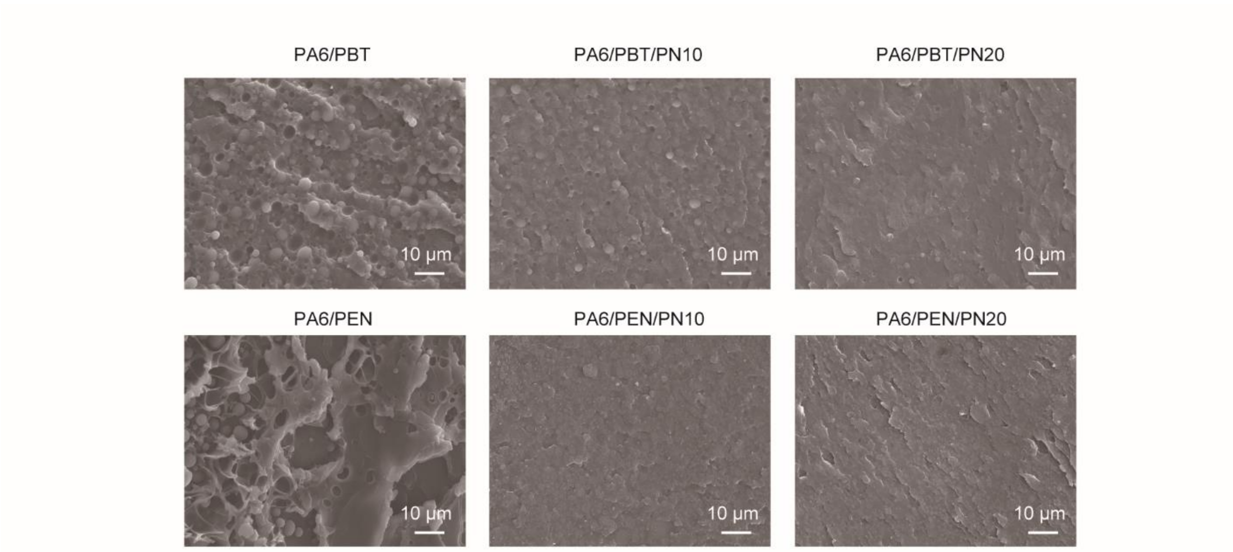

Figure S6. Scanning electron micrographs of the polymer blends. Compatibilization by PN loading is observed in both the PA6/PBT and PA6/PEN blends.

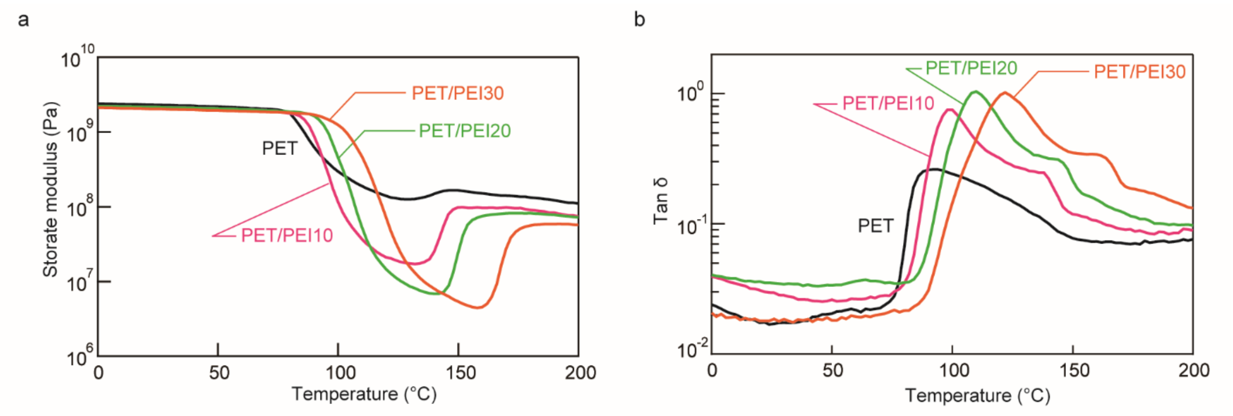

Figure S7. Viscoelastic behavior of homo PET and the PET/PEI blends. Temperature dependencies of (a) the storage modulus and (b) tan δ. PET/PEI blends present a single T_g and their miscibility is confirmed. Re-crystallization behavior is observed in all the specimens, owing to insufficient crystallinity of the prepared specimens.

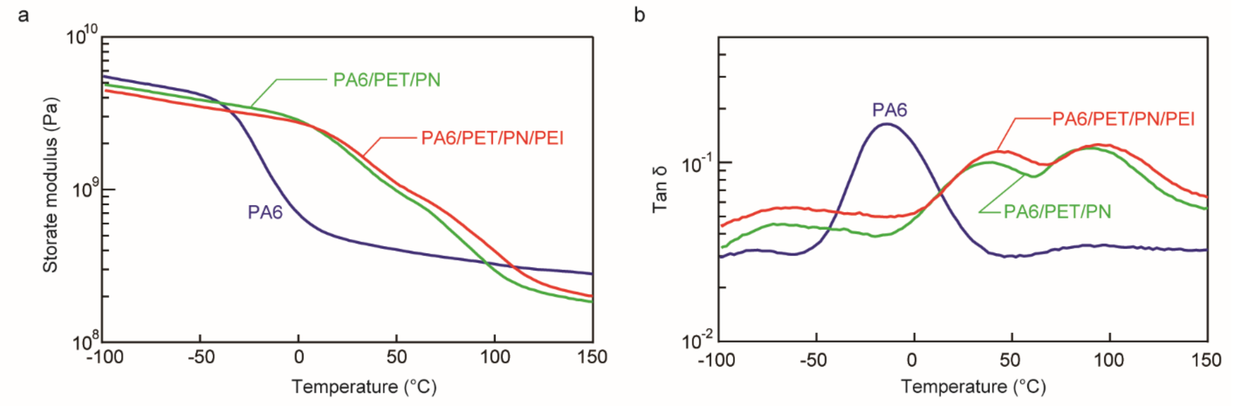

Figure S8. Viscoelastic behavior of the homo PA6, PA6/PET/PN ternary blend, and PA6/PET/PN/PEI quaternary blend in water-absorbed state. Temperature dependencies of (a) the storage modulus and (b) tan δ.

EXPERIMENTAL

Sample Preparation

PA6/PBT Blends

Standard-grade PA6 (A1030BRL, Unitika, Japan), PBT (Duranex 2002, Polyplastics, Japan), and heat-resistant-grade PN (PAPS PN70, Asahi Yukizai, Japan) were employed in this study. All the polymers were vacuum-dried prior to melt mixing. Melt mixing was conducted using an internal mixer (Laboplastomill, Toyo Seiki, Japan) with a cylinder temperature of 250 °C, rotation speed of 100 rpm, and mixing time of 5 min. The sample designations and composition ratios of the prepared blends are listed in Table S1.

PA6/PEN Blends

Standard-grade PA6 (A1030BRL, Unitika, Japan), PEN (Teonex TN-8065S, Teijin Limited, Japan), and heat-resistant-grade PN (PAPS PN70, Asahi Yukizai, Japan) were employed in this study. All the polymers were vacuum-dried prior to melt mixing. Melt mixing was conducted using an internal mixer (Labo Plastomill, Toyo Seiki Seisaku-sho, Japan) with a cylinder temperature of 290 °C, rotation speed of 100 rpm, and mixing time of 5 min. The sample designations and composition ratios of the prepared blends are listed in Table S1.

PET/PEI Blends

Standard-grade PEI (Ultem 1000, Sabic, Saudi Arabia) and injection-molding-grade PET (TRN-0855FF, Teijin Limited, Japan) were employed in this study. All the polymers were vacuum-dried prior to melt mixing. Melt mixing was conducted using a micro compounder (HAAKE Minilab,

Thermo Fisher Scientific, Germany) with a cylinder temperature of 270 °C, rotation speed of 200 rpm, and mixing time of 5 min. The sample designations and composition ratios of the prepared blends are listed in Table S1.

PA6/PET/PN/PEI Blend

Standard grade PA6 (A1030BRL, Unitika, Japan), PEI (Ultem 1000, Sabic, Saudi Arabia), injection-molding grade PET (TRN-0855FF, Teijin Limited, Japan), and heat-resistant grade PN (PAPS PN70, Asahi Yukizai, Japan) were employed in this study. All polymers were vacuum-dried prior to melt mixing. Melt mixing was conducted using an internal mixer (Labo Plastomill, Toyo Seiki Seisaku-sho, Japan) with a cylinder temperature of 270 °C, rotation speed of 100 rpm, and mixing time of 5 min. The sample designations and composition ratios of the prepared blends are listed in Table 1.

Table S1. Sample designations and constituent weight fractions

Sample designation	PA6 (wt%)	PBT (wt%)	PEN (wt%)	PET (wt%)	PEI (wt%)	PN (wt%)
PA6/PBT	50	50				
PA6/PBT/PN10	45	45				10
PA6/PBT/PN20	40	40				20
PA6/PEN	50		50			
PA6/PEN/PN10	45		45			10
PA6/PEN/PN20	40		40			20
PET/PEI10	90				10	
PET/PEI20	80				20	
PET/PEI30	70				30	
PA6/PET/PN/PEI	40			40	10	10

Dynamic Mechanical Analysis (DMA) at Water-Absorbed State

The homo polymers and melt-mixed samples were melt-pressed into rectangular specimens (35 mm × 5 mm × 0.5 mm) using a laboratory press at temperatures of 250, 270, and 290 °C for the polymer blends comprising PBT, PET, and PEN, respectively. The specimens were cooled by a second laboratory press at 150 °C to promote crystallization of the specimens by slow cooling. The specimens listed in Figure S6 were vacuum-dried at 80 °C for 12 h prior to measurement. The other specimens were immersed in distilled water at 23 °C overnight before measurement. DMA was carried out using a dynamic mechanical analyzer (DVA-225, ITK, Japan) in the temperature range -100–200 °C with a heating rate of 5 °C/min and a frequency of 10 Hz.

Scanning Electron Microscopy (SEM)

The cryo-fractured surfaces of the polymer blends were fabricated by fracturing the rectangular specimens (50 mm × 10 mm × 2 mm) in liquid nitrogen. Each fractured surface was sputter-coated with platinum and observed by SEM (S-3600N, Hitachi, Japan) at an accelerating voltage of 15 kV.
